# Selective Loss of Chemokine Receptor Expression on Leukocytes after Cell Isolation

**DOI:** 10.1371/journal.pone.0031297

**Published:** 2012-03-05

**Authors:** Juan C. Nieto, Elisabet Cantó, Carlos Zamora, M. Angels Ortiz, Cándido Juárez, Silvia Vidal

**Affiliations:** 1 Department of Immunology, Institut Recerca Hospital de Sant Pau, Barcelona, Spain; 2 Hospital de Sant Pau, Barcelona, Spain; University of Leuven - Rega Institute, Belgium

## Abstract

Chemokine receptors are distinctively exposed on cells to characterize their migration pattern. However, little is known about factors that may regulate their expression. To determine the optimal conditions for an accurate analysis of chemokine receptors, we compared the expression of CCR2, CCR4, CCR5, CCR6, CXCR3 and CXCR4 on different leukocyte subsets using whole blood (WB) plus erythrocyte lysis and density gradient isolation (Ficoll). Most WB monocytes were CCR2+ (93.5±2.9%) whereas 32.8±6.0% of monocytes from Ficoll-PBMC expressed CCR2 (p<0.001). Significant reductions of CCR6 and CXCR3 on monocytes were also observed after Ficoll isolation (WB: 46.4±7.5% and 57.1±5.5%; Ficoll: 29.5±2.2% and 5.4±4.3% respectively) (p<0.01). Although comparable percentages of WB and Ficoll-PBMC monocytes expressed CCR4, CCR5 and CXCR4, Ficoll isolation significantly reduced the levels of CXCR4 (WB: MFI 5±0.4 and Ficoll: MFI 3.3±0.1) (p<0.05). Similarly to monocytes, CCR2, CXCR3 and CXCR4 were also reduced on lymphocytes. In addition, Ficoll isolation significantly reduced the percentage of CCR4 positive lymphocytes (WB: 90.2±4.5% and Ficoll: 55±4.1%) (p<0.01). The loss of expression of chemokine receptors after isolation of monocytes was not dependent on either the anticoagulant or the density gradient method. It was irreversible and could not be restored by LPS activation or in vitro macrophage differentiation. Experiments tagged with anti-CCR2 antibodies prior to density gradient isolation demonstrated that Ficoll internalized chemokine receptors. The method for cell isolation may alter not only the expression of certain chemokine receptors but also the respective functional migration assay. The final choice to analyze their expression should therefore depend on the receptor to be measured.

## Introduction

The recruitment of leukocytes to inflammation sites is essential for host defense against infectious agents. Chemotactic agents such as chemokines are produced locally and play a crucial role in activating cells during the multistep process of leukocyte accumulation in tissues. Chemokines exert their effects by binding to members of the large family of G-protein-coupled receptors (GPCRs). These chemokine GPCRs signal through heterotrimeric G-proteins consisting of Gαi and Gßc subunits, which in turn regulate a diversity of signal transduction pathways involved in chemotaxis. Chemokine receptors are composed of seven hydrophobic transmembrane domains, an N-terminus outside the cell surface, three extracellular and three intracellular loops, and a C-terminus in the cytoplasmic compartment. Eighteen chemokine receptors have been cloned and they mediate the effects of the more than 50 known chemokines [1].

Some chemokine-receptor mediated signals appear to be redundant while others have central roles in many biological processes, ranging from immunosurveillance to inflammatory responses [2], [3]. Functional experiments have demonstrated that a characteristic expression of chemokine receptors on different cell lineages may have functional significance in terms of placing lymphocytes, monocytes and neutrophils within lymphoid compartments [4]. Although it has been shown that chemokine receptors are exposed on cells and distinct expression profiles characterize the different leukocyte subtypes, little is known about the signals that may regulate their expression.

Interaction of pathogens with migrating cells and engagement of anti-inflammatory cytokine receptors [5] induce switching of chemokine receptor expression. This switch permits cells to home to the inflammatory foci or travel to regional lymph nodes. On the other hand, other cytokines can selectively inhibit expression of chemokine receptors to retain leukocytes at sites of inflammation [6]. To this end, LPS induces both internalization and message degradation of CCR2 [7], [8]. LPS downregulates CCR1 and CCR5 expression in monocytes whereas IL-2 stimulates CCR2 expression [9]. Regulation of CCR2 and CCR5 expression in response to M. tuberculosis has also been described [10], [11].

A distinctive chemokine receptor expression is associated with the state of the cell and consequently its anatomical location. The residence of macrophages is due to the persistence of expression of receptors for inflammatory chemokines such as CCR2 and CCR5. During monocyte maturation, differential signaling mechanisms regulate expression of chemokine receptors [12]. This differentiation significantly increases the number of CCR5 positive cells [13].

Many human diseases are caused by altered expression or mutations in chemokine receptors that lead to inappropriate cell migration [14], [15]. It is therefore critical to determine the expression of chemokine receptors on leukocytes with the utmost sensitivity and accuracy. The favorite analytical method for the detection of chemokine receptors proteins on defined cellular subsets is flow cytometry, which allows a multiparametric analysis of individual chemokine receptor cell expression within larger cell populations.

The precision of detection may depend on the manipulations used to isolate the cells that have to be studied; a small signal induced by subtle manipulations such as temperature shifts or media changes might trigger the internalization of a receptor, rendering the cell apparently negative. To determine the optimal conditions for analyzing the expression of chemokine receptors, we compared the expression of six different chemokine receptors on different cell subsets using variations of staining and cell isolation techniques.

## Materials and Methods

### Samples

Whole blood (WB) samples were collected from healthy donors. Informed consent was obtained and ethical approval for the study was granted by the Institutional Ethics Committee. Samples were processed within one hour after collection and mantained at room temperature. The WB was collected into Vacutainers (BDbiosciences, San Jose, Calif.) containing Sodium-Heparin. For the anticoagulant experiments, blood was collected into separate Vacutainers containing EDTA, Sodium-Heparin, or Acid Citrate Dextrose (ACD).

### Mononuclear cell separation

Human peripheral blood mononuclear cells (PBMC) were separated from peripheral blood of healthy donors by gradient centrifugation on Ficoll-Hypaque (Lymphoprep, AXIS-SHIELD PoCAs, Oslo, Norway) at room temperature. Monocytes were also separated from peripherial blood by Percoll gradient centrifugation (Sigma-Aldrich, St. Louis, Missouri). Two Percoll density gradients were prepared and the first Percoll density gradient (density: 1.087 g/ml) was dispensed on the top of the second Percoll solution (density: 1.077 g/ml). PBS-diluted WB was then layered onto the Percoll gradients without the disturbing the layers and centrifugated at 2000 rpm for 20 min at room temperature. Cells at the first Percoll interface were then collected, washed and resuspended on Staining Buffer (PBS+ bovine serum albumin 0.5%+sodium azide 0.1%).

### WB cell culture

WB was cultured in 5 ml polypropylene tubes (BDbiosciences) by culturing 2.5 ml of heparinized WB in 1 ml of RPMI 1640 medium without 10% Fetal Calf Serum (Biowhittaker), or 2.5 ml of WB in 650 µl of RPMI 1640 medium with 350 µl of lipopolysaccharide (LPS) (0.01 µg/ml) (tlrl-pelps InvivoGen, San Diego, CA, USA) The cultures were maintained at 37°C with 5%CO_2_ until the following day.

### Peripheral blood mononuclear cell cultures

The concentration of isolated PBMC was adjusted to 2×10^6^ cells/ml in 500 µl of RPMI 1640 (Biowhittaker) including 10% Fetal Calf Serum or with 0.01 µg/ml of LPS. Twenty hours later, PBMC were washed, stained (see below) and analyzed by flow cytometry. For macrophage differentiation, Ficoll isolated PBMC were cultured on culture plates (BDbiosciences) in 6 ml of RPMI 1640 (Biowhittaker) including 10% Fetal Calf Serum (Biowhittaker). Seven days later, adherent cells were harvested with EDTA, washed, stained with anti-CD14, anti-CD16, anti-CCR2, anti-CCR5, anti-CXCR3 mAbs and analyzed by flow cytometry.

### WB cell staining

WB was collected on heparine tubes and 100 µl of WB was incubated for 20 min at room temperature in the dark with monoclonal antibodies (Table 1). Red blood cells were then lysed and white cells fixed using TQ-Prep System (Coulter Corp., Miami, Fla) to be analyzed by flow cytometry. For confocal microscopy, whole blood cells were stained with anti-CCR2 PercP/Cy5.5 and anti-CD14 Alexa Fluor 488 (both from Biolegend, San Diego, CA.).We let a drop of cell suspension to dry on a glass slide at room temperature.

**Table 1:** Monoclonal antibodies used for the analysis of chemokine receptor expression.

Antigen	Fluorochrome	Host species and isotype	Manufacturer	Clone
**CD14**	PEDy647	Mouse IgG1	Immunotools	MEM-15
**CD16**	FITC	Mouse IgG1	Immunotools	LNK16
**CCR2**	PE	Mouse IgG2b	R&D Systems	48607
**CCR2**	PerCP/Cy5.5	Mouse IgG2b	BioLegend	TG5/CCR2
**CCR4**	PE	Mouse IgG1	BD	1G1
**CXCR3**	PE	Mouse IgG1	BD	1C6/CXCR3
**CCR5**	FITC	Mouse IgG2b	R&D Systems	45531
**CCR5**	PE	Rat IgG2a	BioLegend	HEK/1/85a
**CCR6**	FITC	Mouse IgG2b	R&D Systems	53103.111
**CCR6**	PE	Mouse IgG2b	BioLegend	TG7/CCR6
**CXCR4**	PE	Mouse IgG2a	BioLegend	12G5
	*PE*	*Mouse IgG2b*	*R&D Systems*	*133303*
	*FITC*	*Mouse IgG2b*	*R&D Systems*	*133303*
***Isotype***	*PE*	*Mouse IgG1*	*BD*	*MOPC-21*
***Controls***	*PE*	*Mouse IgG2b*	*BioLegend*	*MG2b-57*
	*PE*	*Mouse IgG2a*	*BioLegend*	*MOPC-173*

### Peripheral blood mononuclear cell staining and confocal microscopy imaging

PBMC were washed with staining buffer (PBS+bovine serum albumin 0.5%+sodium azide 0.1%) and resuspended in 100 µl of staining buffer. Monoclonal antibodies (see Table 1) were added to the cells, and the tubes were incubated for 20 min at room temperature in the dark. Samples were then washed and resuspended in 300 µl of PBS+paraformaldehyde 0.4% to be analyzed by flow cytometry. For the receptor internalization experiments WB cells were incubated with anti-CCR2-PE (R&D, Weisbaden, Germany), for 20 min in the dark, cells were then separated by density gradient Ficoll, stained with anti-CD14-PEDy647 (Immunotools, Friesoythe, Germany). Samples were analyzed and compared by Flow Cytometry. For confocal microscopy, PBMC were isolated as above and stained with anti-CCR2 PercP/Cy5.5 and anti-CD14 Alexa Fluor 488 (both from Biolegend, San Diego, CA.).We let a drop of cell suspension to dry on a glass slide at room temperature. To assess antibody location, images were obtained with a LEICA TCS SP5 laser scanning confocal microscope. Argon laser with excitation wavelengths of 488 nm was used for Alexa 488 label analysis and Hene laser with excitation wavelengths of 633 nm was used for PercP/Cy5.5 label analysis.

### Flow cytometry analysis

Surface expression of chemokine receptors was analyzed on monocyte subpopulation (gated according to side scatter parameter and CD14 positive expression), on macrophages differentiated from peripheral blood monocytes (CD14+ and CD16+ cells), and on lymphocytes and neutrophils (gated according to forward and side scatter parameters). These analyses were performed on a Beckman Coulter F500 cytometer. The percentage of positive cells (% cells) and Mean Fluorescence Intensity (MFI) of each individual marker were calculated using EXPO™ 32 MiltiCOMP Software (Beckman Coulter).

### Chemotaxis Assay

The chemotaxis of monocytes from WB or PBMC in response to recombinant human CCL2 (Immunotools) and fMLP (Sigma-Aldrich, St. Louis, Missouri) was measured across 3 µm pore-size cell culture inserts incorporating polyethylene terephthalate membranes in 24-well companion plates (Millicell cell culture inserts) (Millipore, Billerica, MA). Complete medium (RPMI-1640 including 10% Fetal Calf Serum), supplemented or not with recombinant human CCL2 (0.2, 2 and 20 ng/ml) or fMLP (10^−8^ M and 10^−6^ M), was placed in the lower chamber of the plate. Peripheral blood from healthy volunteers was collected into a Sodium-Heparin tube, WB was centrifugated at 1750 rpm for 5 min at room temperature, supernantant was then descarted and cells were resuspended on the same volume of culture medium and placed into culture inserts (total volume of 100 µl per insert). Ficoll-isolated cells were adjusted at 2×10^6^ cells/ml in culture medium and 100 µl were placed into culture inserts. The plates were then incubated for 4 hr at 37°C (5% CO2). Cells that had crossed the membrane were collected from the lower chamber, pelleted down, resuspended in 150 µl of culture medium and incubated for 20 min at room temperature with 10 µl of anti-CD66b FITC, 10 µl of anti-CD3 PE mAb and 10 µl of anti- CD14 PEDy647 mAbs (Immunotools). After washing in PBS, red blood cells were lysed, and white cells fixed using TQ-Prep System (Beckman Coulter) to be analyzed by flow cytometry. Monocytes were gated on the basis of forward- and side-scatter, and the percentage of CD14+ cells was determined.

### Statistics

Differences between conditions were analyzed using a parametric test (Student's t-test). Values were expressed as percentages or mean fluorescence intensity (MFI)±SEM deviation. P values<0.05 were considered significant.

## Results

### Expression levels of chemokine receptors on leukocytes from WB and Ficoll-isolated PBMC

To study the expression of chemokine receptors on leukocytes, we first compared the expression levels of CCR2, CCR4, CCR5, CCR6, CXCR3 and CXCR4 on leukocytes from whole peripheral heparinized blood (WB) and PBMC isolated by density gradient Ficoll (Ficoll-PBMC).

**Figure 1:**
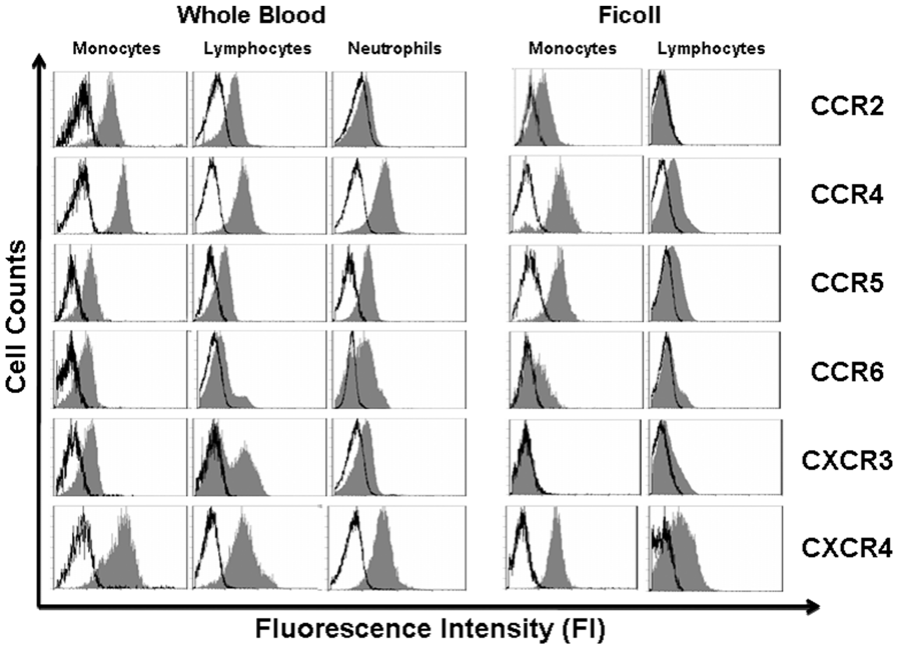
Levels of chemokine receptor expression on leukocytes from WB and Ficoll isolated PBMC. Representative experiment with leukocytes from healthy donors *(n = 12)*. Monocytes were stained with anti-CD14 mAbs and lymphocytes and neutrophils were selected by FS and SS parameters. Leukocytes were also stained with monoclonal antibodies for chemokine receptor CCR2, CCR4, CCR5 CCR6, CXCR3 and CXCR4. Profile of expression was assesed by flow cytometry (isotype control **□** , chemokine receptor ▪).

The expression of chemokine receptors on monocytes from WB and Ficoll-PBMC was analyzed after gating on CD14+ cells (Table 2 and Fig. 1). CCR4 and CCR5 expression on monocytes from WB and Ficoll-PBMC was similar (CCR4 on WB MFI: 6.5±0.2, on Ficoll MFI: 4.4±0.3; CCR5 on WB MFI: 3.6±0.4, on Ficoll MFI: 2.9±0.2 respectively). In contrast, whereas most WB monocytes were CCR2+ (93.5±2.9%) less than 30% of monocytes from Ficoll-PBMC expressed CCR2 on the surface (32.8±6.0%)(p<0.001). Monocytes became CXCR3 negative after Ficoll isolation (5.4±4.3%) (p<0.01) and expressed less CXCR4 than monocytes from WB (WB MFI: 5±0.4 Ficoll MFI: 3.3±0.1 respectively) (p<0.05). CCR6+ monocytes were also significantly reduced after Ficoll isolation (46.4±7.5% in WB and 29.5±2.2% in Ficoll) (p<0.01) (Table 2 and Fig. 1).

**Table 2:** Chemokine receptor expression on leukocytes from peripheral blood.

		WHOLE BLOOD			FICOLL	
		Monocytes	Lymphocytes	Neutrophils	Monocytes	Lymphocytes
		Mean ± SEM	Mean ± SEM	Mean ± SEM	Mean ± SEM	Mean ± SEM
**CCR2**	% Cells	93.5±2.9	48±6.9	23.5±7.7	32.8±6.0***	4.8±2.0**
	MFI	4.7±0,.2	1.3±0.1	1.5±0.2	1.6±0.7***	0.9±0.3**
**CCR4**	% Cells	93.7±1.2	90.2±4.5	73.1±10.7	88.9±1.1	55±4.1**
	MFI	6.5±0.2	3.8±0.6	6.1±0.9	4.4±0.3	2.3±0.2
**CCR5**	% Cells	87.2±6.6	58±6.8	89.9±2.8	86.5±4.7	52.7±5.7
	MFI	3.6±0.4	2.1±0.4	2.8±0.3	2.9±0.2	1.4±0.07
**CCR6**	% Cells	46.4±7.5	39.3±9.5	65.7±9.0	29.5±2.2**	25.2±2.1
	MFI	1.5±0.1	1.2±0.1	1.9±0.2	1.1±0.3	1.6±0.2
**CXCR3**	% Cells	57.1±5.5	56.1±7.3	15.4±4,.2	5.4±4.3**	9.8±5.0**
	MFI	1.3±0.03	2.8±0.2	2.4±0.3	0.3±0.8	1.6±0.05
**CXCR4**	% Cells	95.2±0.4	86.4±6.2	89.8±4.7	94.5±6.2	41.7±1.5**
	MFI	5±0.4	2.8±0.6	3.04±0.5	3.3±0.1*	1.9±0.1

MFI: Mean Fluorescence Intensity.

* p<0.05,

** p<0.01,

*** p<0.001.

An analogous analysis was performed on gated lymphocytes. Similarly to monocytes, after Ficoll isolation, the percentage of lymphocytes expressing CCR2 and CXCR3 was significantly reduced (CCR2: 48±6.9% in WB and 4.8±2.01% on Ficoll-PBMC, p<0.001 and CXCR3: 56.1±7.3% in WB and 9.8±5.07% in Ficoll PBMC, p<0.01). No differences in the expression levels of CCR5 and CCR6 were observed between lymphocytes from WB and Ficoll-PBMC. A comparable percentage of lymphocytes from WB and Ficoll-PBMC was CCR6+ in concordance with the reported CCR6 expression on memory T cells and B lymphocytes [16]. Significantly fewer Ficoll-isolated lymphocytes were CCR4 and CXCR4 positive than WB lymphocytes (CCR4: WB 90.2±4.5% Ficoll: 55±4.1% p<0.01 and CXCR4: WB: 86.4±6.2 Ficoll: 41.7±1.5 p<0.01).

Chemokine receptor expression was also analyzed on WB neutrophils. Neutrophils were identified and distinguished from lymphocytes, monocytes and DCs by virtue of their high FSC and SSC [17].

The expression of CCR4, CCR5 and CCR6 on WB neutrophils was similar to that on WB monocytes. However, fewer WB neutrophils than WB monocytes were CCR2 and CXCR3 positive (CCR2+ monocytes: 93.5±2.9, CCR2+ neutrophils: 23.5±7.7; CXCR3+ monocytes: 57.1±5.5, CXCR3+ neutrophils: 15.4±4.2).

This chemokine receptor downregulation induced by Ficoll separation could be either the consequence of epitope destruction or masking, or antigen removal from cell surface. To discern between these two alternatives, leukocytes from Ficoll-PBMC were stained with two monoclonal antibodies recognizing different extracellular epitopes on CCR2, CCR5 or CCR6 proteins. None of the different monoclonal antibodies were able to detect these proteins on the leukocytes, suggesting that the receptors were removed from the cell surface (data not shown).

As expected, each leukocyte subset has a particular profile of chemokine receptors on their cell surface. Some chemokine receptors showed significant differences between mononuclear cells isolated by Ficoll and WB. Whereas the expression of certain chemokine receptors was significantly reduced after Ficoll isolation, leukocytes retained the expression of other chemokine receptors. It is also striking that after Ficoll isolation each leukocyte subset underwent a particular reduction.

### Effect of anticoagulant and separation methods on the chemokine receptor expression

**Figure 2:**
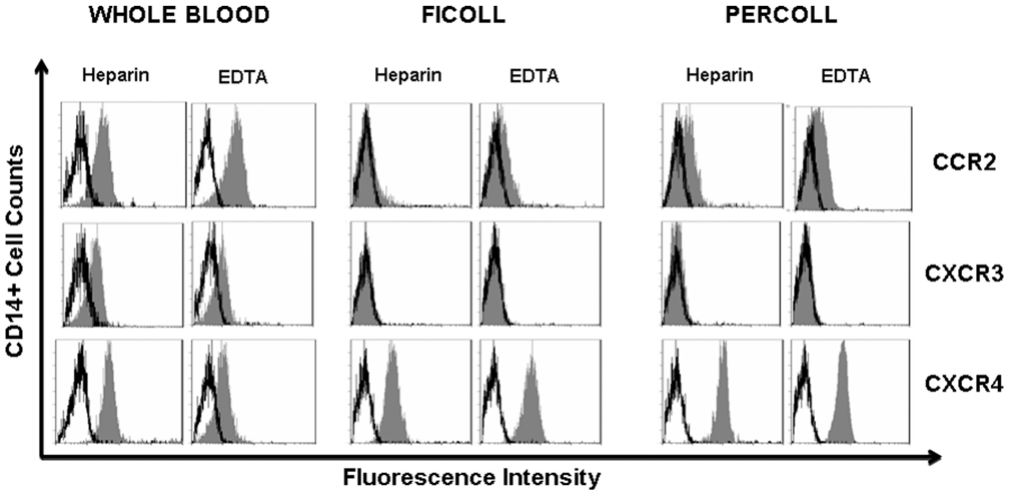
Effect of anticoagulant and the cell separation method on monocyte chemokine receptors expression. WB, Ficoll and Percoll isolated PBMC from healthy donors (*n = 3*) were collected in Vacutainer tubs containing sodium heparine or EDTA. Monocytes were CD14+ gated and CCR2 (clone 48607), CXCR3 and CXCR4 expression were determined. The results of a representative cytometric analysis were shown *(n = 5)* (isotype control **□** , chemokine receptor ▪).

To evaluate the influence of anticoagulants, we compared the influence of heparin and Ca2+ chelators such as EDTA on the chemokine receptor expression of Ficoll-isolated monocytes. Monocytes collected in heparin- or EDTA- WB expressed similar levels of CCR2 and CXCR3. A significant reduction of the CXCR4 expression was observed on the EDTA-WB monocytes (MFI 5.43±0.35 in heparin WB and MFI 1.2±0.04 in EDTA WB p<0.05). The use of EDTA instead of heparin did not prevent CCR2 and CXCR3 downregulation after Ficoll isolation (CCR2: MFI 3.8±0.8 in heparin WB, 3.0±0.07 in EDTA WB, Ficoll MFI 1.6±0.6 in heparin, MFI 1.0±0.07 in EDTA. CXCR3: MFI 1.3±0.7 in heparin WB, 1.4±0.3 in EDTA WB, Ficoll MFI 0.3±0.6 in heparin, MFI 0.5±0.01 in EDTA). The downregulation of chemokine receptors on the Ficoll isolated monocytes was not influenced by the type of anticoagulant into which the peripheral blood was collected (Fig. 2).

Chemokine receptor expression was then assessed on monocytes enriched by a discontinuous density Percoll gradient from peripheral blood collected in Vacutainer tubes with sodium heparin or EDTA. CCR2 and CXCR3 expression levels on Percoll and Ficoll-isolated monocytes were comparably downregulated, regardless of the anticoagulant and method of separation used (Fig 2). In contrast, CXCR4 expression was not downregulated. The expression on monocytes from EDTA and heparin WB, Ficoll and Percoll-isolation was comparable (Ficoll: MFI 4.9±0.9 in heparin MFI 5.4±0.05 EDTA; Percoll: MFI 5.2±0.7 in heparin MFI 5.4±0.06 in EDTA).

### Chemokine receptor expression on seven-day cultured monocytes and overnight LPS culture

**Figure 3:**
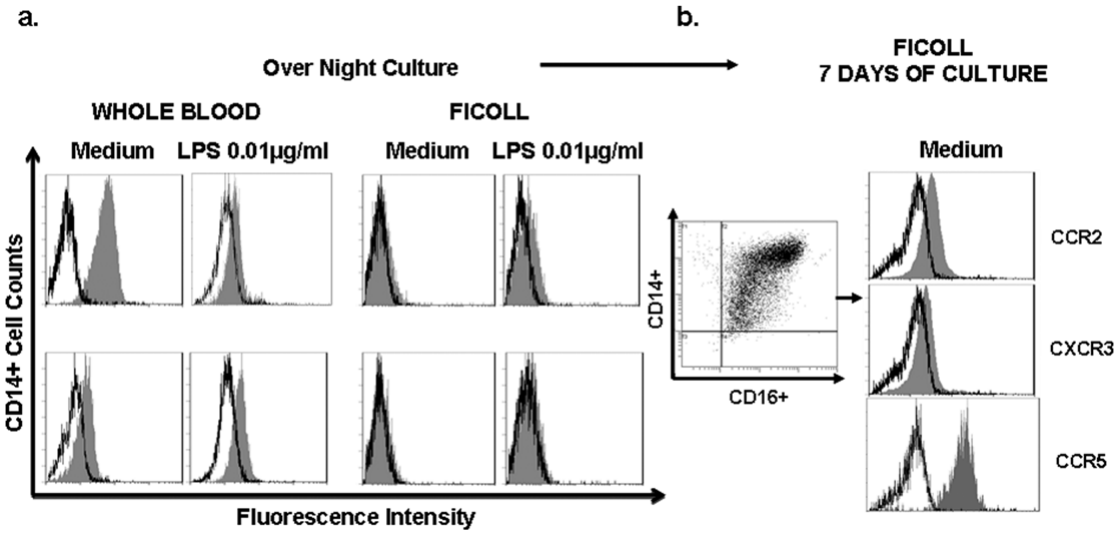
Chemokine receptor expression on LPS and seven-day cultured monocytes. The results of a representative experiment are shown. a) Monocytes from WB or monocytes from Ficoll were culture 20 h in medium or LPS (0.01 µg/ml) and stained with anti-CCR2-PE or anti-CXCR3-PE. b) Monocytes from Ficoll were cultured in medium and seven days later, cells were harvested and stained with anti-CCR2-PE, anti-CXCR3-PE and anti-CCR5-PE (isotype control **□** , chemokine receptor ▪).

Differentiation of monocytes into macrophages has been directly associated with changes in CD16 and CCR expression levels. After monocyte in vitro differentiation, we observed CCR5 increases similar to previously reported [13]. CCR2 and CXCR3 expression were therefore examined on 7-day-cultured monocytes from Ficoll isolated peripheral blood. Neither cell differentiation nor the consequent adherent conditions modified the expression of Ficoll-downregulated chemokine receptors (CCR2: MFI 4.3±0.5 in medium WB, Ficoll seven-day of culture: MFI 2.6±1.3, CXCR3: MFI 1.6±0.1 in medium WB, Ficoll seven-day of culture: MFI 2.1±0.8) (Fig. 3).

Further experiments were performed to determine whether chemokine receptor expression could be restored by lipopolysacharide (LPS), a prototypic maturation signal. While WB overnight cultures did not alter CCR2 or CXCR3 expression on monocytes, the addition of 0.01 µg/ml of LPS to WB cultures significantly reduced CCR2 but not CXCR3 expression (CCR2: medium 91.8±2.5% LPS 42.4±4.6%). Overnight culture of Ficoll-PBMC monocytes with medium or LPS 0.01 µg/ml did not prevent the downregulation of CCR2 and CXCR3 (Fig. 3).

### Determination of the fate of down regulation of CCR2 on monocytes from Ficoll

**Figure 4:**
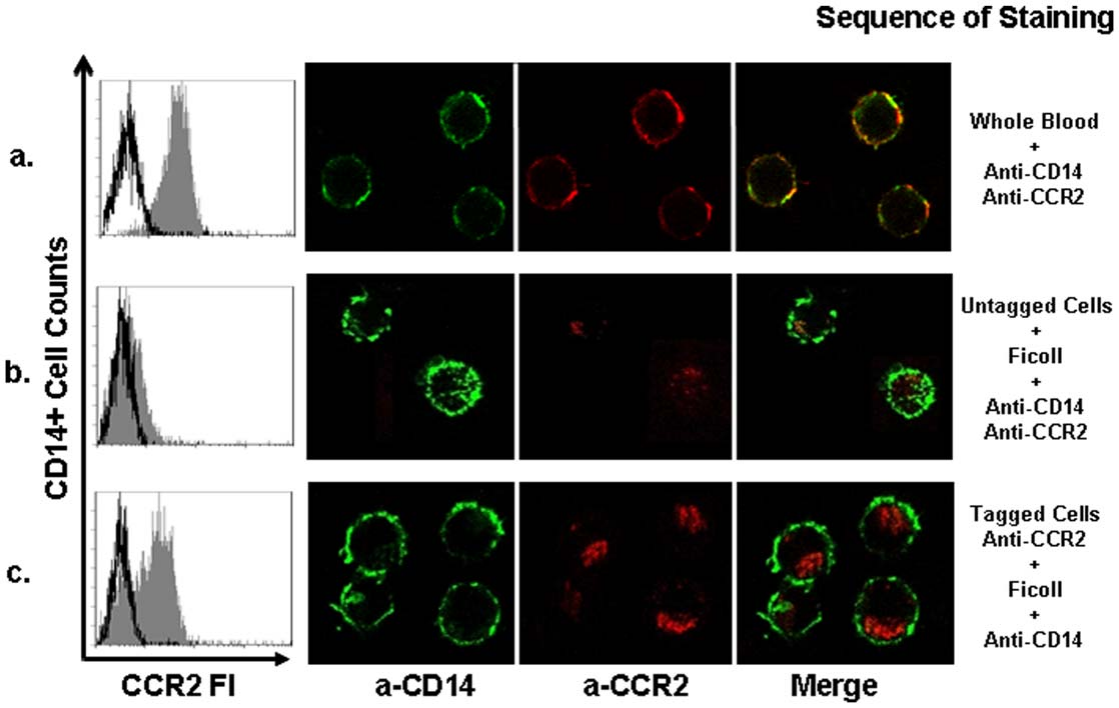
Determination of fate of CCR2 downregulation on monocytes from Ficoll. CCR2 expression was assessed after Ficoll isolation of untagged and tagged-CCR2 monocytes. Internalization of the complex was examined from one single layer of Z stacks by confocal microscopy. The results of a representative experiment are shown: a) CCR2 expression on monocytes from WB tagged with CCR2 prior to gradient separation; b) CCR2 expression on untagged monocytes after Ficoll isolation and the subsequent staining of CCR2; c) CCR2 expression in CCR2 tagged monocytes after Ficoll isolation, red signal within monocytes represents the CCR2-complex internalization (no aditional anti CCR2-PE was added to the cells after isolation with Ficoll); (isotype control **□** , chemokine receptor ▪).

To determine the fate of chemokine receptors on the cell surface we analyzed the endocytosis of a monoclonal antibody recognizing the extracellular domain of CCR2 to follow the trafficking of the surface CCR2. CCR2 molecules on monocytes from WB were tagged with PE-conjugated anti-CCR2 monoclonal antibodies prior to separation. PBMC were then separated by Ficoll gradient from tagged and untagged WB cells. To gate on monocytes, tagged PBMC were stained with anti-CD14 and untagged cells were stained with anti-CD14 plus anti-CCR2. Flow cytometry analysis showed that monocytes with tagged CCR2 molecules prior to Ficoll separation were CCR2 positive (Ficoll MFI: 2.5±0.09) similarly to monocytes from WB (MFI 4.7±0.2). In contrast, untagged monocytes simultaneously stained with anti-CD14 and anti-CCR2 antibodies after Ficoll separation were negative (MFI 1.5±0.2). Confocal microscopy showed that CCR2 expression was located in the cytoplasm of tagged cells whereas WB tagged with PE-conjugated anti-CCR2 monoclonal antibodies prior to separation showed cell surface location (Fig. 4). The fluorescence intensities of CCR2 positive cells of tagged cells stained with anti-CD14 and tagged cells stained with both anti-CD14 and anti-CCR2 antibodies were similar (data not shown). Although monocytes from Ficoll-PBMC and WB are in possession of an equivalent number of CCR2, only those molecules on WB monocytes were accessible to the monoclonal antibodies. These results demonstrated that standard density gradient isolation procedures internalize chemokine receptors. It is unclear whether this internalization targets chemokine receptors to the endosomal/lysosomal compartment.

### Monocyte migration capacity towards CCL2 depends on the isolation method

**Figure 5:**
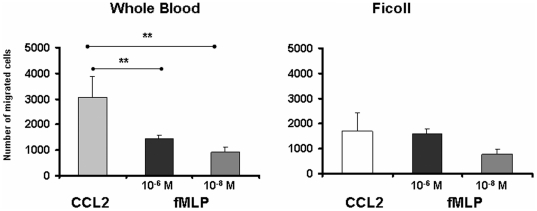
Migration capacity of monocytes from WB and Ficoll PBMC towards CCL2. WB diluited 1∶5 in medium (100 µl per well) or Ficoll PBMC (2×10^6^ cells/ml per well) were subjected to a 4 h-chemotaxis assay towards CCL2 or fMLP *(n = 5)*. The cells that had migrated into the lower chamber were collected and stained with anti-CD14-PEDy647 and analyzed by flow cytometry. Results are expressed as the mean of the number of migrated cells ± SEM.

To directly test the influence of the isolated method on the chemokine receptor function, we compared the chemotactic responses of monocytes from WB or isolated by Ficoll to CCL2. For this purpose, we measured the chemotaxis capacity of monocytes from WB or isolated by Ficoll from five healthy donors (n = 5) in presence of CCL2 at 2 ng/ml We observed that the number of monocytes isolated by density gradient Ficoll (1701±581) exhibited a significantly reduced chemotaxis toward CCL2 (p<0.05) compared to monocytes from WB (3082±806) (Fig. 5). To further analyze the extend of the migratory defect of Ficoll-isolated monocytes regardeless of different interfering factors in whole blood and MNC culture, we compared the migration to fMLP (a standard chemoattractant) [18] and CCL2 . The chemotactic response of WB monocytes to CCL2 was significantly higher than to 10^−6^ M fMLP (2.08 fold, p<0.01). However, the chemotactic response of Ficoll-isolated monocytes to CCL2 was comparable to fMLP (1.05 fold). Regardless of different soluble factors or cell interactions that could take place in WB and MNC 4 h culture, the down-regulation of monocyte CCR2 expression by Ficoll may be associated with a decreased ability of monocytes to migrate towards CCL2.

## Discussion

Our results showed that the expression analysis of specific chemokine receptors is compromised after standard density separation. Flow cytometry along with erythrocyte-lysed fresh blood introduced minimal artifacts on the immunophenotyping. On the other hand, density separation reduced not only the positive levels of chemokine receptor expression quantified by MFI but even rendered cells negative for posterior staining and functional assays. The loss of expression of these chemokine receptors on the cell surface was irreversible and it could not be restored by activation or differentiation strategies.

Several authors have reported that sample preparation has a significant impact on the quality of the staining of surface antigens on major leukocyte subsets. [19]. In particular, processing methods such as lysis of WB or Ficoll isolation yield different results in flow cytometric analysis of CD34+ cells. Staining of Ficoll-Hypaque-separated cells was essentially negative, whereas a clear positive population was evident with the lysed preparation [20]. Detection of integrin and C3b receptors was also dependent on the manipulations used to isolate monocytes [21].

In regard to chemokine receptors, a recent report on the staining of WB followed by lysis gave the best sensitivity for CCR2, CCR5, and CXCR3 on lymphocytes [22]. Consequently, plastic adherence, elutriation, Ficoll or Percoll gradient followed or not by culture in the presence of growth factors have possibly originated repeated discrepancies in the detection of chemokine receptors on peripheral blood monocytes [13], [23]. Some results could be excluded because they lack the proper isotype controls for the marker setting. Another major source of discrepancies between reports may have come from significant individual variations or batch-to-batch effect of reagents. Depending on the epitope recognized by each antibody, variable percentages of positive cells have thus been detected [13].

Several hypotheses may account for the reduced sensitivity after a density gradient. Ficoll separation has been shown to lead to the loss of specific subsets of cells [24]. However, this explanation is not applicable in our experiments since an equivalent percentage of CD14+ cells was detected in all the tubes (data not shown). The selective reduction in the sensitivity to certain chemokine receptors could also be due to the monoclonal antibody used in the staining assay. However, comparative studies with monoclonal antibodies from other manufacturers were performed to exclude this possibility.

An explanation for the reduced detection could be that the antigen was modulated from the cell surface or blocked by components of the Ficoll. Our tagged results showed that CCR2 was detected intracellularly in Ficoll separated monocytes. The receptor was likely missing due to endocytosis rather than cleavage or blocking. It is unclear whether the addition of sodium azide prior to manipulation of cells could have prevented any active internalization of membrane receptors initiated by the cell manipulation. In these circumstances, cell incubation at 37°C following isolation would have allowed chemokine receptors in the endosome compartment to recycle to the surface [25], [26], [27]. However, we did not perceive the return to original levels after longer cultures at 37°C in the absence of sodium azide.

Some of the expression changes, such as lower CCR2 and CXCR4 after density gradient separation, could uncover an unspecific stimulation of the cells by Ficoll components. Indeed, endotoxin contamination has been associated with Na(+)-heparin Vacutainer tubes, affecting assay measurements [28]. A recent test indicated that multiple lots of sodium heparin tubes have variable background levels of endotoxin (range <0.01 to 1.04 EU/ml) [17]. However, if this was the case, WB and Ficoll separated monocytes would have similarly been in contact with contaminated tubes and consequently, both conditions would have comparably internalized the chemokine receptors. The immunostaining of WB and Ficoll PBMC was performed within 2 h of blood donation to avoid an uncontrolled monocyte differentiation and its consequent change in antigen presentation.

It is striking that Ficoll induced changes did not provoke a general internalization of all chemokine receptors on monocytes. This could be a reflection of the different speed for internalization of chemokine receptors [29]. Previous reports have also obtained concordant results on T lymphocytes [22].

Although the staining of WB cells prior to red blood cell lysis is the recommended protocol, this method is not always appropriate for functional assessments. The receptor disappearance induced by density gradient isolation results in unresponsiveness of these cells to their respective ligands. However, it should be noted that the current experiments cannot completely rule out the influence of other factors in the migratory ability of whole blood cells. The present observations are relevant for validating multiple reports that have isolated monocytes previously to the analysis of the migration patterns. In conclusion, there is no optimal method for the determination of all chemokine receptors on leukocytes and the most precise method will depend on which receptor is being measured.
